# A Bibliometric Analysis of the HCV Drug-Resistant Majority and Minority Variants

**DOI:** 10.3390/ijerph22111670

**Published:** 2025-11-03

**Authors:** Omega Mathew Immanuel, Olaoluwa Tolulope Fabiyi, Kuat P. Oshakbayev, Gulzhan Abuova, Aliya Konysbekova, Sreenu B. Vattipally, Syed Ali, Syed Hani Abidi

**Affiliations:** 1 Department of Biomedical Sciences, Nazarbayev University School of Medicine, Astana 010000, Kazakhstan; omega.immanuel@nu.edu.kz (O.M.I.); olaoluwa.fabiyi@nu.edu.kz (O.T.F.); syed.ali@nu.edu.kz (S.A.); 2 University Medical Center, Nazarbayev University, Astana 010000, Kazakhstan; kuat.oshakbayev@umc.org.kz; 3 Department of Infectious Diseases and Dermatovenereology, South Kazakhstan Medical Academy, Shymkent 160019, Kazakhstan; dr.abuova@gmail.com; 4 Clinic of Hepatology, Gastroenterology and Nutrition, Astana 010000, Kazakhstan; aliyakonysbekova@gmail.com; 5 MRC-University of Glasgow Centre for Virus Research, University of Glasgow, Glasgow G61 1QH, UK; sreenu.vattipally@glasgow.ac.uk

**Keywords:** HCV, drug-resistant variants, mutation, interferon, DAAs, next-generation, sequencing

## Abstract

Background: In recent decades, research on Hepatitis C Virus (HCV) drug-resistant variants has expanded; however, critical gaps remain in our understanding of global contributions, emerging trends, and future research directions. Here, we present a bibliometric analysis to understand the research themes and trends in research related to HCV drug-resistant variants published between 1999 and 2025. Methods: Publications related to HCV drug-resistant variants published between 1999 and 2025 were searched on the Web of Science and Scopus databases. Publication metadata and content-based data were extracted and analyzed using Bibliometrix and VOSviewer for keyword co-occurrence plot and cluster analysis. Results: The analysis of 653 articles revealed a clear paradigm shift, driven by the introduction of direct-acting antivirals (DAAs), which led to a significant surge in annual publications, peaking between 2014 and 2018. This shift in focus led to an emphasis on DAA efficacy, resistance mechanisms, and advanced genotyping. The United States was the most productive country, with the highest number of publications (*n* = 134) and citations (*n* = 6458). The University of São Paulo was the most productive institution (*n* = 40), while Antimicrobial Agents and Chemotherapy published the highest number of articles in this field (*n* = 40). Susser S. was the most productive researcher. Collaboration networks were found to be predominantly centered in high-income countries. Analysis of studies on minority variants showed that most studies originated from Europe and the United States, identifying low-frequency resistance-associated substitutions (RASs) such as A156V, D168V, Y93H, and S282T, with prevalence ranging from <1% to 35%, which were frequently associated with viral breakthrough and reduced treatment response. Conclusions: The field successfully transitioned to the DAA era, but research output and collaboration networks were primarily driven by high-income countries, leaving a critical gap in data from Low- and Middle-Income Countries (LMICs). Closing this gap by integrating LMIC data is the next essential step to ensure global elimination strategies are effective for all countries from different income strata.

## 1. Introduction

Hepatitis C Virus (HCV) infection is a major global health challenge, responsible for 50 million infections and 24,200 deaths globally as of 2022 [1]. HCV is a bloodborne virus that primarily affects the liver, causing hepatitis, cirrhosis, and hepatocellular carcinoma [2]. The virus’s high mutation rate, shaped by error-prone RNA-dependent RNA polymerase, high replication rate, and host- and drug-selection pressure, drives its genetic diversity, which leads to the emergence of drug-resistant variants/quasispecies that can negatively affect the treatment efficacy [3,4,5].

HCV research has undergone a temporal evolution, closely tied to the significant changes in HCV diagnostics and treatment strategies. Treatment has evolved from the interferon era to a revolution with first-generation DAAs, and currently to the pan-genotypic DAA era, which can achieve >95% cure rates across all major genotypes with a high barrier to resistance [6,7,8]. Interferon-based therapies were once the mainstay of treatment for HCV [9]. However, current HCV treatment relies on direct-acting antivirals (DAAs) targeting viral proteins essential for replication [10]. The first-generation DAAs, the NS3/4A protease inhibitors, were associated with a limited spectrum and efficacy, and significant resistance [6,7], leading to the introduction of second-generation DAAs, NS3/4A, NS5B polymerase, and NS5A inhibitors, which have broader genotype coverage and higher efficacy, but with substantial resistance [8,11]. Currently, the pan-genotypic third-generation DAAs, such as glecaprevir/pibrentasvir and sofosbuvir/velpatasvir, are used in the management of HCV because they are more effective (achieve a sustained virologic response rate of ≥95%) and have a high barrier to resistance [12,13]. Regimens are tailored based on genotype, prior treatment, cirrhosis status, and resistance profile [14,15]. Pan-genotypic DAAs have played a crucial role in reaching the WHO HCV global elimination target, which aims for a 90% diagnosis rate and an 80% treatment rate among infected people, resulting in an 80% decrease in new infections and a 65% decline in mortality [16].

HCV drug resistance arises from resistance-associated substitutions (RASs) occurring in the NS3/4A, NS5A, or NS5B regions of the viral genome, which can reduce drug binding or activity [5,13,17,18,19]. RASs can exist naturally (baseline RASs) or emerge during treatment due to selective pressure from suboptimal treatment (e.g., monotherapy, poor adherence) [8]. Baseline RASs are detected in 5–20% of untreated patients, varying by genotype [20,21]. Baseline RASs are more common in genotypes 1a and 3, and treatment-emergent RASs, such as NS3 Q80K, complicate retreatment due to cross-resistance [22,23].

Drug resistance has significantly affected the global HCV elimination goal, especially in resource-limited settings with limited access to resistance testing [24,25]. Drug-resistant variants have significant public health implications, with both the dominant (majority) and low-frequency (minority) variants contributing to treatment failure, particularly in treatment-experienced or cirrhotic patients [12]. Therefore, resistance testing is essential to reduce the burden of resistant variants [26].

Advances in DAAs and molecular typing have improved outcomes; however, ongoing research is needed to improve access to HCV diagnostics and treatment, and address the issue of antiviral resistance [24]. A shift from Sanger sequencing, which detects dominant RASs but misses low-frequency variants (<20% of the viral population [27]), to next-generation sequencing (NGS) that detects low-frequency RASs (1–5%) with high sensitivity, has significantly improved resistance profiling [21,28]. Additionally, whole-genome deep sequencing technologies have also aided in identifying genotypes/subtypes for treatment selection, as well as in transmission and phylogenetic studies [27,29]. Although the transition from interferon therapy to pan-genotypic DAAs has reduced the risk of drug resistance to antiviral treatment [10], surveillance for viral quasispecies remains critical for predicting outbreaks and managing hard-to-treat populations, such as males (reportedly having lower cure rates than females), genotype 3 (more aggressive genotype) patients, and those with cirrhosis [14,30].

Bibliometrics has been applied in several fields of science [31], as it is an essential tool for assessing and analyzing the output of scientists, collaborations, and research trends [32]. This tool can be used to identify which countries and institutions have made the most significant contributions, which journals publish the most literature, and to determine the current state of research on HCV drug-resistant variants. While studies before 2010 primarily focused on HCV drug resistance mutations, very few have examined minority variants and their impact on treatment outcomes. Consequently, no bibliometric analysis thus far has reported the evolution of research themes, trends, and global contributions in this field. Therefore, this bibliometric study aimed to analyze articles on HCV drug-resistant variant research to identify research trends and hotspots and connect the findings to the implementation of global elimination strategies.

## 2. Methodology

The Web of Science (WoS) database, which is widely used for bibliometric analysis [33], was sourced for this study. This database indexes over 12,000 high-impact journals across various disciplines, making it a reliable source for analyzing research output, collaborations, and trends, compared to the Scopus and PubMed databases [34,35]. To further enhance our search results, a complementary search was also performed in the Scopus database. We searched Scopus and WOS for literature published from 1999 to 2025, a period selected to capture the temporal dynamics of HCV drug resistance research, ranging from the use of interferon to modern DAAs. All results from the search query were exported in plain text format for screening and data analysis.

### 2.1. Search Strategy

This study utilized a combination of Medical Subject Headings (MeSH) and non-MESH terms to enhance article retrieval from Scopus and WoS using the following search string: (((ctTS = (“hepatitis C virus” OR “HCV” OR “hepacivirus”) AND TS = (“drug resistan*” OR “antiviral resistan*” OR “resistance-associated substitution*” OR RAS)) AND TS = (“majority variant*” OR “minority variant*” OR “low-frequency variant*” OR “high-frequency variant*” OR quasispecies)) OR TS = (“low abundance” OR “high abundance” OR “low frequency” OR “high frequency”)) OR TS = (variant* OR mutation* OR substitution*), and TITLE-ABS-KEY ((TS = (“hepatitis C virus” OR “HCV” OR “hepacivirus”) AND (“drug resistant” OR “antiviral resistant” OR “resistance-associated substitution” OR RAS)) AND (“majority variant” OR “minority variant” OR “low-frequency variant*” OR “high-frequency variant*” OR quasispecies)), for WoS and Scopus, respectively.

### 2.2. Screening Protocol and Criteria

The initial search yielded 4712 records. Through a systematic screening process conducted independently by two researchers, 15 duplicate articles and 3913 items comprising review articles, conference proceedings, editorials, letters, and book chapters were excluded. The remaining 768 articles were manually screened based on titles, abstracts, and keywords, with a focus on relevance to “HCV drug-resistant majority and minority variants.” Where necessary, we performed full-text evaluations to assess eligibility.

The inter-rater agreement, calculated using the kappa statistic, was found to be 0.90, suggesting excellent inter-rater agreement. Any disagreements between the reviewers were resolved through arbitration by the senior author, resulting in a final dataset comprising 653 articles for further analysis. 

**Figure 1:**
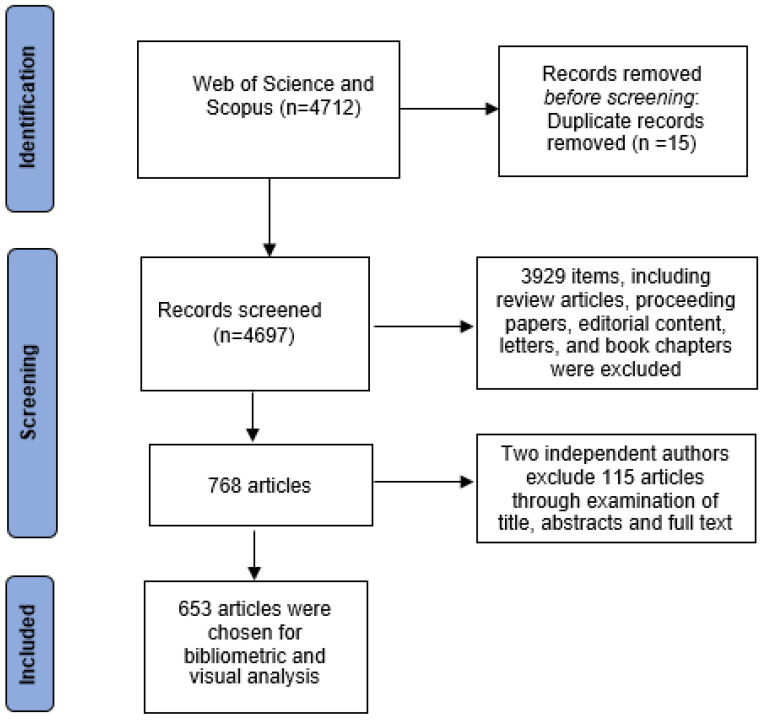
The process of literature retrieval and selection based on the PRISMA framework.

The literature retrieval and selection process is summarized in Figure 1, following the PRISMA guidelines [36].

### 2.3. Data Analysis

The 653 eligible articles were exported in plain text format. Two complementary bibliometric tools, Bibliometrix (v3.2.1) in R Studio 4.2.3 [37,38] and VOSviewer (v1.6.19) [39,40], were employed to, respectively, analyze annual publication trends, country and institutional contributions, journal distribution, and citation metrics; and keyword co-occurrence mapping, thematic cluster identification, and density visualization of research landscapes.

Additionally, co-authorship networks were generated from bibliographic data to identify patterns of collaboration. Normalized citation metrics were used to account for the varying citation windows of the publications.

### 2.4. In-Depth Analysis of Top-Cited Studies on HCV Minority Variant

To characterize fundamental research on HCV drug-resistant minority variants, a supplementary analysis was conducted on the most-cited articles on HCV minority variants from the final dataset. We systematically selected the top 10 articles with the highest total citation count (TC) and extracted information regarding sequencing method, minority variant detected, prevalence, and country of the corresponding author.

## 3. Results

### 3.1. Analysis of Publication Output

**Figure 2:**
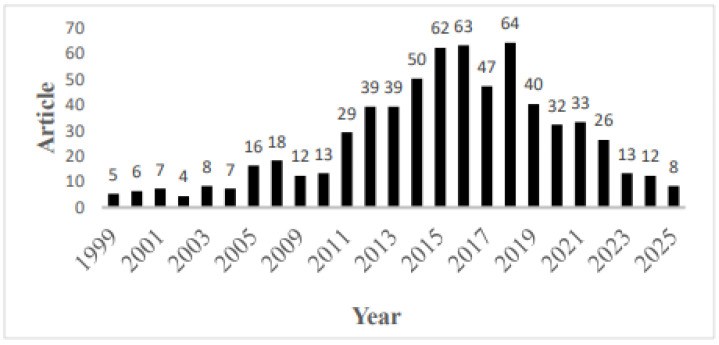
Annual scientific production (Annual percentage growth rate 1.82).

Analysis of 653 articles on HCV drug-resistant variants, from 1999 to 2025, suggested that the annual publication output rose steadily from 1999, peaking at 64 articles in 2018, followed by a gradual decline. The lowest output was recorded in 2002 (*n* = 4). The mean annual growth rate was 1.82% over the entire period (Figure 2).

Notably, the period from 2014 to 2018 marked a surge phase, with an average output of 57.2 publications. However, by 2024, the publications had decreased to approximately one-fifth of the peak volume (Figure 2).

### 3.2. Analysis of Countries and Institutions

The top ten countries contributing to HCV drug-resistant variants research included a mix of developed nations, the USA, Japan, Italy, Spain, Germany, France, and Denmark, and newly industrialized/developing economies (Brazil, Iran, and China; Table 1). Among these, Germany stood out with the highest level of international collaboration (MCP ratio = 40.62).

**Figure 3:**
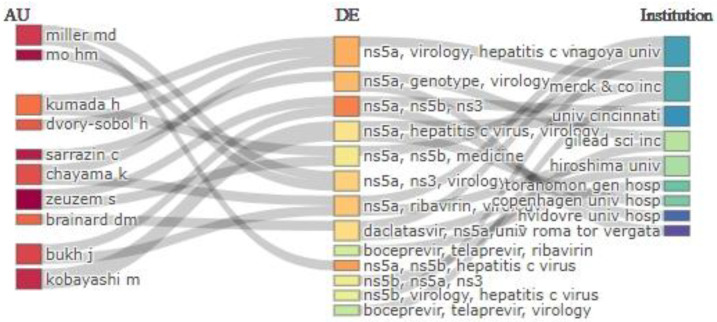
A three-field map showing the research landscape, linking authors, keywords, and their affiliated institutions. The grey lines show the flow (connections) between the authors (AU), keywords (DE), and institutes, while the thickness of the line indicates the strength/frequency of association.

The Bibliometrix three-field plot, which examines the relationships among institutions, authors, and keywords [37], mapping the top 10 institutions, 15 authors, and 15 keywords, revealed that the research landscape is structured around specialized universities and private research institutions, with a key focus on NS5A, NS5B, and NS3 inhibitors (Figure 3).

**Figure 4:**
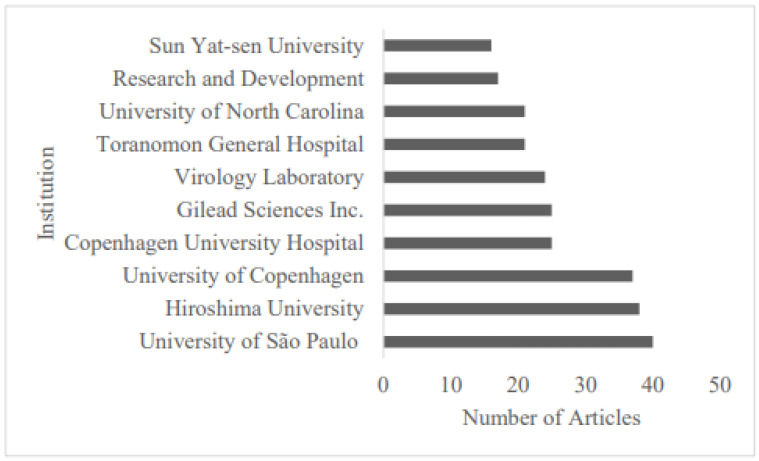
Top 10 institutions by production count.

Analysis of publishing institutes revealed that most articles were from universities and public health organizations. Notably, the University of São Paulo (Brazil) produced the highest institutional output during the review period (*n* = 40; Figure 4).

### 3.3. Analysis of Journals

The 653 selected articles were published across 174 journals, highlighting the broad dissemination of research on HCV drug-resistant variants. Table 2 ranks the top ten most active journals in this field by publication volume. Among them, the journal *Antimicrobial Agents and Chemotherapy* published the most articles in the field (*n* = 46). However, the *Journal of Virology* dominated as the journal with the most cited articles (Total Citations = 1285, g-index = 35.85).

### 3.4. Analysis of Citations

#### 3.4.1. Top-Cited Papers on HCV Drug Resistance

Table 3 presents the ten most globally cited papers on HCV drug-resistant variants. The highest-cited study was “*Characterization of resistance to the protease inhibitor boceprevir in hepatitis C virus–infected patients*” by Susser S et al. [17], published in Hepatology in 2009, with 264 total citations, averaging 15.5 citations per year, while the article “*NS5A resistance-associated substitutions in patients with genotype 1 hepatitis C virus: Prevalence and effect on treatment outcome*” by Zeuzem S et al. [19] had the highest annual citation rate (19.3 citations/year). When adjusted for citation window differences using Normalized Total Citations (NTC), the article “*Prevalence of resistance-associated substitutions in HCV NS5A, NS5B, or NS3 and outcomes of treatment with Ledipasvir and Sofosbuvir*” by Sarrazin C et al. [6] ranked highest with NTC of 9.10.

#### 3.4.2. Country-Level Citations

The U.S. dominated among the top 10 countries, accumulating 6458 citations and an average article citation of 45.48 (Table 4). The USA was followed by Japan, with 1884 citations, and Germany, with 1125 citations (all developed countries).

### 3.5. Analysis of Collaborative Networks

**Figure 5:**
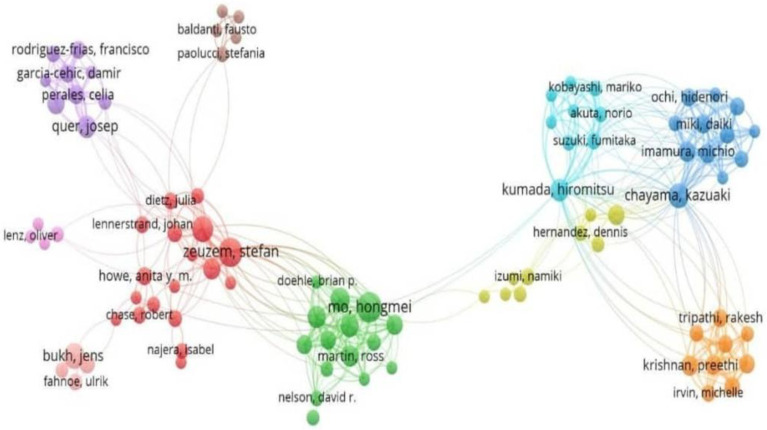
Analysis of co-authorship network: Each node represents an author, the size of which corresponds to the number of publications by that author, and the colour indicates the research cluster or collaborative group to which the author belongs [red (Central European collaboration group), green (U.S. and European Clinical/Virology Research Group), blue (Japanese research network), orange (emerging or peripheral collaborators), pink (Southern European collaboration), brown (peripheral European contributors), and yellow (bridging authors)].

Analysis of co-authorship networks revealed distinct collaborative clusters representing both national and international research groups (Figure 5). The red cluster, one of the central and most influential networks, was primarily composed of European collaborators, especially centered in Germany, with Zeuzem acting as a key investigator linking researchers from clinical and virological backgrounds. The blue cluster represents a strong within-country network in Japan, indicating high national collaboration and consistent co-authorship among Japanese researchers. Similarly, the green cluster represents a collaborative effort between U.S. and European institutions, highlighting cross-continental research partnerships. The yellow cluster connects the U.S. and Japan; the pink cluster includes Italy and Spain; the orange cluster represents emerging collaborators from India and Denmark; and the brown cluster includes smaller European subgroups, such as Belgium and the Netherlands. The orange cluster depicts emerging or regional collaborations, while the pink cluster represents a relatively independent Southern European network that maintains limited links with the central European core. The brown cluster comprises peripheral European contributors, and the yellow cluster contains bridging authors who connect the green and blue clusters, thereby facilitating international collaboration between the U.S. and Japan. The dense interconnections suggest strong national collaboration within Germany and Japan, as well as consistent co-authorship across multiple publications.

### 3.6. Cluster Analysis and Temporal Trends in HCV Drug Resistance Research

**Figure 6:**
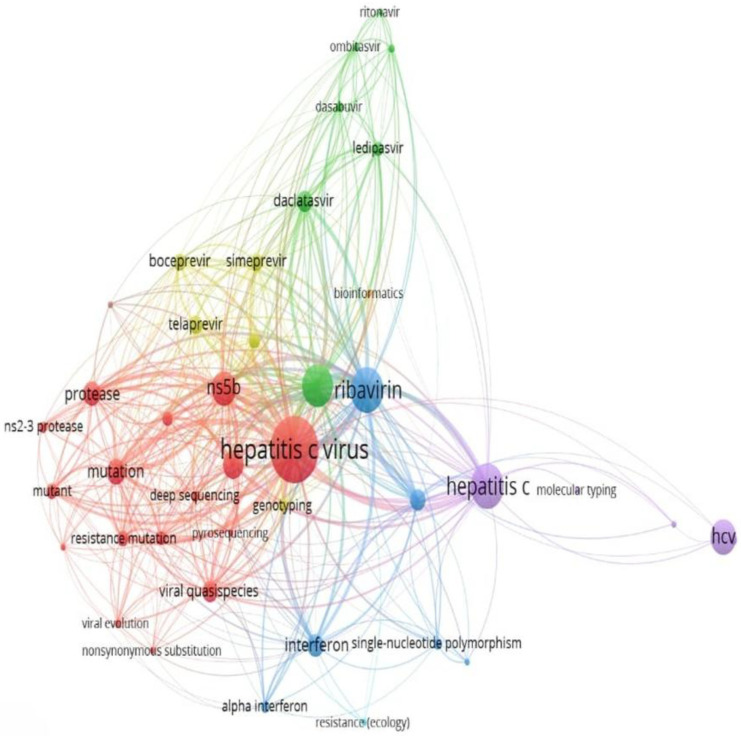
Keywords cluster analysis of HCV drug-resistant variants (occurrence ≥ 5 times). The colors represent the clusters, and the darker the color, the higher the frequency of keyword occurrence; the same color indicates similar research directions. The red cluster focuses on viral evolution and resistance; the green cluster relates to DAAs; the blue cluster centers on traditional treatments and genetic factors; the purple cluster highlights molecular typing and genotyping, and the yellow cluster represents bioinformatics and sequencing tools.

The keyword cluster analysis results for terms with at least five occurrences, generated using VOSviewer, identified the following key terms reported in publications: viral evolution and resistance, deep sequencing, Hepatitis C virus, mutation, NS5B, RASs, viral quasispecies, protease, resistance mutation, viral quasispecies, deep sequencing, sofosbuvir, ledipasvir, daclatasvir, ombitasvir, ritonavir, simeprevir, interferon, alpha interferon, ribavirin, single-nucleotide polymorphism, and resistance, hepatitis C, HCV, molecular typing, genotyping, bioinformatics, pyrosequencing, next-generation sequencing, boceprevir, and telaprevir (Figure 6).

**Figure 7:**
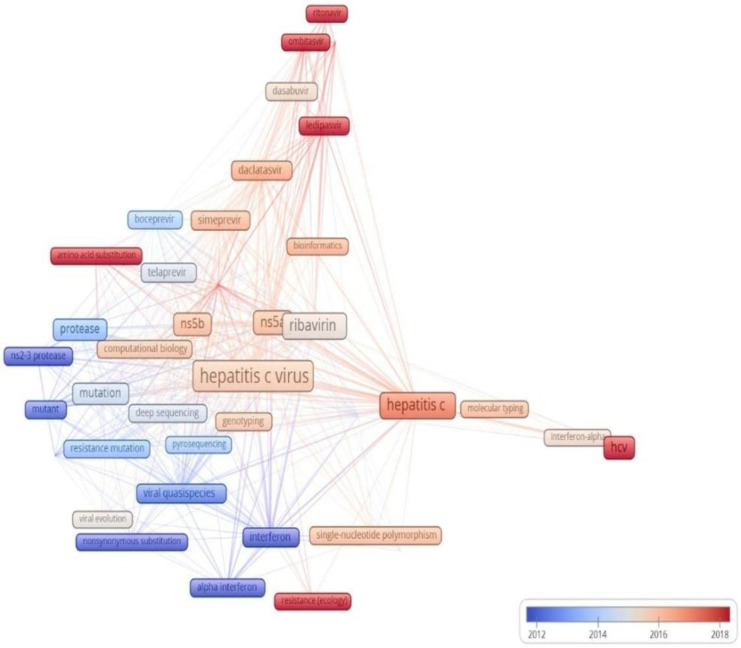
Temporal keywords co-occurrence analysis of HCV drug-resistant variants (occurrence ≥ 5 times). The color gradient (purple → red) indicates time progression, with purple representing older trends and red indicating recent developments.

An analysis of the temporal co-occurrence of keywords in HCV drug resistance research revealed a timeline of major themes from 1999 to 2025, displayed through a color gradient from purple to red. Early research (purple and blue) mainly focused on interferon therapy, ribavirin, and viral genotyping, representing the pre-DAA period. As the field progressed (green to yellow), attention shifted toward DAAs, such as protease and polymerase inhibitors. The latest phase (orange to red) highlights NGS, RASs, and pan-genotypic regimens, emphasizing the increasing use of molecular surveillance for monitoring antiviral resistance (Figure 7).

### 3.7. Analysis of Key Trends in Minority Variants Research

To understand the evolution, focus areas, and research dynamics within studies specifically addressing HCV minority variants, an in-depth analysis was conducted on the most highly cited papers in this field. The characteristics of the ten most-cited studies that explicitly focused on low-frequency variants are summarized in Table 5. The analysis reveals that the most influential studies predominantly originated from European countries (Belgium, Spain, France, Italy, England) and the United States, with Verbinnen, T being the most cited author (59 citations). Earlier studies (2002–2005) relied on clonal and Sanger sequencing, while later studies (2010–2018) used deep sequencing and ultra-deep sequencing platforms. The most commonly detected minority variants include F43S, A156G, A156V, D168V, and D168A in the NS3 protease gene; S282T in the NS5B polymerase gene; and T54S, V36A, V170A, Q80K, V36L, V55A, V36M, and Y93H in the NS5A region, with prevalence ranging from <1–35%. The analysis revealed that the detection of these RASs was consistently linked to reduced treatment efficacy, lower cure rates, and higher rates of treatment failure (Table 5).

## 4. Discussion

This bibliometric analysis examines research trends, publication and citation patterns, and key trends in the field of HCV drug-resistance research, with a focus on minority variants, from 1999 to 2025. By analyzing publications over this period, this study not only identifies temporal publication dynamics and key trends but also highlights persistent knowledge gaps within this field.

### 4.1. Major Publication Trends During the Pre-DAA and DAA Eras and Their Importance for Global Health

*Pre-DAA era*: From 1999 to 2009, research output was low but consistent (~10–30 papers/year), and most studies focused on interferon–ribavirin therapy, its (limited) efficacy, and significant side effects, diverting focus from resistance mechanisms [9]. Studies during this period mainly focused on resistance in non-responders, as viral escape variants were a secondary concern compared to broader treatment limitations [2].

*DAA-era*: Between 2010 and 2014, publication rates increased significantly, reaching ~60 papers/year by 2014, driven by the introduction of first-generation protease inhibitors including telaprevir, boceprevir, and danoprevir. Although these drugs improved sustained virological response (SVR rates), they were associated with high resistance rates, likely due to key mutations such as NS3/4A-R155K, NS3/4A-T54, and NS3/V36M, which posed significant clinical challenges such as immune evasion, high virologic relapse, and failure rates, etc. [47,58,59]. Publication output peaked at ~60–62 papers/year between 2015 and 2017, coinciding with the advent of second-generation DAAs, including NS5A inhibitors (e.g., ledipasvir, daclatasvir) and sofosbuvir-based regimens. These therapies improved treatment efficacy but introduced new resistance issues, particularly NS5A-associated mutation Y93H, prompting research into retreatment strategies [42,60,61]. From 2021 to 2025, publications related to drug resistance declined to ~30–40 papers/year, likely coinciding with the success of pan-genotypic regimens such as glecaprevir/pibrentasvir, which achieved SVR rates >95% with minimal resistance [13]. The use of pan-genomic regimens, for instance, sofosbuvir/velpatasvir/voxilaprevir, in patients who have failed prior NS5A-inhibitor treatment, helps overcome viral resistance, achieving cure rates of over 95% even in treatment-experienced patients who previously exhibited resistance to earlier-generation DAAs [13]. The pan-genomic regimens also prevent the emergence of resistant viral strains and reduce treatment failures [13].

The fall in research output (by ~80%) from 2018 to 2024–2025 may also reflect a shift in research focus from drug resistance, due to the success of pan-genotypic DAAs, to HCV elimination goals, point-of-care diagnostics, and addressing underserved populations [15,62]. This change in research focus suggests that highly effective pan-genotypic DAAs have rendered resistance a less critical clinical issue [8,12,13,15,23]. At the same time, the global push for elimination shifted attention and resources toward public health implementation, access, and equity [24,25,26,62]. Additionally, technological advancements such as next-generation sequencing (NGS) have fundamentally transformed the understanding and study of HCV drug resistance, viral diversity, and treatment outcomes [27,28].

The global distribution and accessibility of third-generation pan-genotypic DAAs represent a significant milestone in the worldwide effort to eliminate HCV, owing to their high efficacy (SVR > 95%) across all genotypes and patient subgroups, and their ability to markedly reduce the risk of treatment failure, viral breakthrough, and the emergence of new resistant strains [8,13,23]. Notably, the widespread rollout of sofosbuvir/velpatasvir and glecaprevir/pibrentasvir has already led to a sharp decline in resistance-related treatment failures in both clinical trials and real-world settings [13,14,23], underscoring their crucial role in controlling global HCV drug resistance. Despite this success, global inequities in access remain a substantial challenge. In LMICs, high drug prices, limited diagnostic infrastructure, and supply chain barriers restrict access to third-generation DAAs, threatening to sustain reservoirs of resistant HCV strains [24,25,63,64]. Thus, there is a need to systematically address these issues to ensure that HCV elimination goals can be successfully and equally achieved in all parts of the world [65,66].

### 4.2. Major Publishing Countries and Significant Challenges for Global HCV Research

The analysis of major countries contributing to HCV resistance research showed that the United States had the most research output (134 articles), followed by Japan (92 articles) and China (54 articles). This reflects robust research infrastructure in the U.S. and Japan [67] and China’s growing focus on infectious diseases [68,69]. Notably, Germany demonstrated the highest level of international collaboration with an MCP ratio of 40.62. Universities and public health institutions, notably the University of São Paulo, the University of Copenhagen, and Hiroshima University, were key contributors, providing region-specific resistance data that may have played a key role in optimizing DAA treatments [70,71,72,73,74]. The three-field visualization mapping authors, keywords, and their affiliated institutions identified five distinct clusters, corresponding to established research teams from Germany, Denmark, Sweden, the U.S., and Japan, as well as emerging or peripheral collaborators alongside bridging authors from India and Italy. These research clusters focused mainly on “NS5A,” “NS5B,” and “NS3,” suggesting that the most significant research activities were concentrated on the viral targets of DAAs, reflecting the field’s focus on the mechanisms of antiviral resistance, as discussed in foundational papers on DAA efficacy and resistance [8,13,17]. Furthermore, the collaboration network also revealed that the research is primarily driven by collaborations among academic hospitals, universities, and pharmaceutical entities. This underscores the translational nature of the work, bridging basic virology with drug development.

While the collaboration network indicates that key contributions come from institutions in developed countries, it also reveals a critical gap in contributions from institutions in LMICs. This disparity threatens the equity of global elimination efforts, as highlighted in recent analyses [24,25]. For example, Alenzi [25] reported that in LMICs, factors such as out-of-pocket expenses, lower income, and lack of awareness contribute to reduced access to healthcare services and lower treatment uptake, especially among disadvantaged populations [25]. These inequities can be addressed through multifaceted interventions, such as providing free treatment (for example, through support from government or government-private partnership), reducing the prices of DAAs, expanding healthcare coverage, and strategic collaboration between industry, academic, community, and non-profit researchers to support the production and distribution of generic medicines in LMICs [25,63,65,66]. These supports can also enhance screening, linkage to care, and treatment adherence, thereby narrowing the disparity gap and accelerating progress toward the global hepatitis C elimination goals.

Highly cited works include Susser et al. [17], with 264 citations for characterizing NS5A resistance-associated substitutions (RASs), and Zeuzem et al. [19], with a high annual citation rate (19.3 citations/year) for shaping treatment guidelines. Lin et al. [41,45] provided foundational insights into NS3/4A resistance mechanisms. The U.S. led in citations (6458), driven by NIH funding and pharmaceutical involvement, while Japan and Europe contributed region-specific data. China’s increasing output may suggest its growing influence and contribution to the research. A gradual decline in annual citations (2.013 fewer citations per year) may signal a maturing field as DAA therapies stabilize. It is important to note that the environment of funding cuts could significantly impact HCV research, among other fields [75,76].

### 4.3. Key Trends in HCV Drug Resistance and Its Impact on Global HCV Research

Keyword analysis revealed thematic clusters reflecting the evolution of HCV research. The cluster of “interferon” and “ribavirin” reflects the pre-DAA era, characterized by low SVR rates, and “IL28B polymorphism” may indicate the influence of host genetics on viral control [77,78,79]. Similarly, keywords such as “sofosbuvir,” “ledipasvir”, and “daclatasvir” mark the DAA revolution, though resistance persisted across genotypes [80,81]. Additionally, keywords such as “mutation”, “viral quasispecies”, “NS5B”, “protease”, “viral evolution and resistance”, “direct-acting antivirals (DAAs)”, “molecular typing and genotyping”, “bioinformatics”, and “sequencing” highlight resistance under antiviral pressure, with deep sequencing enabling detection of low-frequency mutations [21,29].

Overall, the trend analysis shows a shift from interferon-based studies and early sequencing to DAAs and modern typing methods. HCV research has evolved from addressing interferon limitations (1999–2009), to characterizing resistance with first-generation protease inhibitors (2010–2014), to NS5A resistance during the DAA peak (2015–2017), and finally to focusing on elimination and access with pan-genotypic regimens (2021–2025). The transition to NGS has enhanced resistance detection, treatment optimization, and elimination monitoring [82,83]. This advancement has significant public health implications for the elimination of HCV. By identifying low-frequency minority variants that often cause treatment failure, NGS enables clinicians to select the most effective regimens from the outset (for example, baseline mutations) [25,84]. This detailed genomic data also strengthens national surveillance, helping to track emerging resistance and transmission clusters, which is vital for effective elimination programs [85,86]. Ultimately, NGS facilitates a “precision medicine” approach that improves cure rates, even in complex cases [87,88]. Collectively, these gains make the fight against HCV more efficient and bring the WHO’s 2030 elimination target within closer reach [25,89].

### 4.4. Research on HCV Minority Variants and Their Impact on Global HCV Elimination

The most cited research on minority variants was conducted in European countries (Belgium, Spain, France, and Italy) and the United States. This aligns with the broader bibliometric finding that high-income countries have driven the research agenda. Technologically, a clear evolution was seen from the early use of clonal and Sanger sequencing, which have limited sensitivity for low-frequency variants, to the adoption of more sensitive deep and ultra-deep sequencing methods in later studies. This shift was pivotal, as it unveiled a previously hidden layer of viral diversity (minor variants) including F43S, A156G, D168V/A in the NS3 region, S282T in NS5B, and key NS5A variants like Y93H, V36A/M/L, and T54S. These variants, which are known to confer resistance to various DAA classes, can pre-exist at low frequencies (<1% to 35%) and expand under selective drug pressure, leading to treatment failure [90,91,92].

The introduction of highly effective pan-genotypic DAAs has markedly reduced the risk that a single pre-existing minority variant will lead to treatment failure [13]. Despite this progress, concerns remain for specific, hard-to-treat populations. These include patients with advanced cirrhosis or prior treatment failures, who harbor more diverse and complex viral quasispecies [14,23]; individuals infected with genotype 3, where RASs like Y93H can still compromise the efficacy of certain regimens [18,23]; and male patients, who exhibit lower cure rates, potentially due to a complex interplay of factors [30].

In this context, NGS is a critical tool for ensuring therapeutic success. Critically, deploying NGS in LMICs is a strategic necessity for global elimination. It facilitates the characterization of region-specific viral genetics, ensuring treatment strategies are effective against local strains and optimizing the use of available therapies in resource-limited settings [24,25].

We anticipate certain limitations of this study. The main limitation relates to the database selection, as the analysis was limited to articles listed in WoS and Scopus. These are the two most trusted sources of bibliometric data [93]. However, WoS is widely used for bibliometric analysis [33], as a more reliable database compared to Scopus and PubMed databases [34,35]. Secondly, in this bibliometric analysis, all selected studies were published in English. It is important to mention that our search queries were not restricted by language; the search databases may have a bias towards English-language publications, as no non-English studies were identified [93]. However, for the current bibliometric analysis, which aims to provide key research trends in HCV drug resistance research, the impact of excluding relevant non-English studies may have been minimal [94,95]. This limitation can be addressed in a future systematic review on the same subject, where each included study carries substantial weight in the final evidence synthesis [96].

## 5. Conclusions

This bibliometric analysis provides a comprehensive overview of the evolving research landscape on HCV drug-resistant variants from 1999 to 2025. The findings show a clear shift from interferon failure to optimizing DAA efficacy through the NGS-based surveillance of minority variants. However, this progress was found to be geographically uneven, as the HCV research in this sphere, along with collaborations, was concentrated in high-income countries. This is concerning and highlights strategic vulnerability in the global elimination agenda. The most pressing gap seems no longer technical, but geographical and inequitable. Current and future research efforts should be focused on ensuring that pan-genotypic treatment is globally available and accessible to all patients. Furthermore, continued monitoring of minority variants, primarily through highly sensitive NGS-based assays, is required to ensure that the third-generation DAAs stay effective and can be effectively implemented as a strategy to eliminate HCV globally.

## Figures and Tables

**Table 1:** Publication output and international collaboration patterns by corresponding authors’ countries. The table shows the top 10 countries contributing the most articles, their frequency (Freq), and the SCP (Single Country Publications), MCP (Multiple Country Publications), and MCP ratio (proportion of a country’s publications produced through international collaboration).

	Country	Articles	Freq	SCP	MCP	MCP Ratio
1	USA	134	0.205	113	21	15.67
2	Japan	92	0.141	88	4	4.35
3	China	54	0.087	48	6	11.11
4	Italy	41	0.063	36	5	12.20
5	Spain	32	0.049	28	4	12.50
6	Germany	32	0.049	19	13	40.62
7	Brazil	31	0.048	29	2	6.45
8	France	18	0.028	17	1	5.56
9	Denmark	15	0.023	13	2	13.33
10	Iran	14	0.021	13	1	7.14

**Table 2:** Ranking of leading journals by publication volume and citation impact.

	Articles	h-Index	m-Index	g-Index	TC	Number of Articles
1	*Antimicrobial Agents and Chemotherapy*	299	0.152	47.24	2232	46
2	*Antiviral Therap* *y*	94	0.047	26.23	688	40
3	*Journal of Viral Hepatitis*	112	0.056	23.39	547	32
4	*Journal of Medical Virology*	160	0.081	23.54	554	31
5	*PLOS One*	467	0.233	22.43	503	30
6	*Antiviral Research*	157	0.079	25.08	629	21
7	*Scientific Reports*	347	0.173	18.57	345	18
8	*Viruses*	141	0.07	12.41	154	18
9	*Journal of Virology*	330	0.168	35.85	1285	17
10	*Virology Journal*	104	0.052	18.63	347	16

Keyword: m-index: Annualized h-index; g-index: The number of top papers whose total citations equal at least the square of that number; TC = total citation.

**Table 3:** Ranking of influential articles by total and normalized citation count. The table shows the first author and the year of publication, DOI, Total Citations (TC), and Normalized Total Citations (NTC).

S.No	First-Author, Year	DOI	TC	TC per Year	NTC
1	Susser S, 2009 [17]	10.1002/hep.23192	264	15.5	4.82
2	Lin C, 2004 [41]	10.1074/jbc.M313020200	259	11.8	3.31
3	Fridell RA, 2010 [42]	10.1002/hep.24594	204	13.6	4.92
4	Lenz O, 2010 [43]	10.1128/AAC.01452-09	200	12.5	4.86
5	Tong X, 2006 [44]	10.1016/j.antiviral.2005.12.003	196	9.8	4.09
6	Lin C, 2005 [45]	10.1074/jbc.M506462200	191	9.1	3.67
7	Sarrazin C, 2016 [6]	10.1053/j.gastro.2016.06.002	180	18.0	9.10
8	Svarovskaia ES, 2014 [46]	10.1093/cid/ciu697	175	14.6	7.63
9	Zeuzem S, 2017 [19]	10.1016/j.jhep.2017.01.007	174	19.3	7.51
10	Romano KP, 2012 [47]	10.1371/journal.ppat.1002832	173	12.4	5.16

**Table 4:** Total citations per country.

S.No	Country	Articles	Average Article Citation
1	USA	6458	45.48
2	Japan	1884	19.83
3	Germany	1125	36.29
4	Italy	858	19.07
5	Spain	808	25.25
6	China	721	11.27
7	France	535	24.32
8	Belgium	523	37.36
9	Denmark	468	31.20
10	UK	462	24.32

**Table 5:** Characteristics of the top-cited articles focusing on HCV minority drug-resistant variants.

	First-Author, Year	Sequencing Method	Variants Detected	Prevalence	Country	Citations	Clinical Outcome
**1**	Verbinnen, T; 2010 [48]	Clonal sequencing and Deep sequencing	F43S; A156G; A156V; D168V; D168A	<1%	Belgium	59	NS
**2**	Puig-Basagoiti, F, 2005 [49]	Clonal and Sanger sequencing	NS	4–35%	Spain	51	Reduced treatment efficacy
**3**	Soler, M, 2002 [50]	Clonal and Sanger sequencing	NS	21%	France	51	NS
**4**	Di Maio, VC, 2014 [51]	NS	NS	NS	Italy	50	Reduced treatment efficacy
**5**	Gregori, J, 2013 [52]	Pyrosequencing	NS	0.70%	Spain	47	NS
**6**	Soria, ME, 2018 [53]	Ultra-deep sequencing	NS	10%	Spain	28	NS
**7**	Le Pogam, S, 2012 [54]	Clonal sequencing	S282T	24.7%	USA	25	Reduced treatment efficacy
**8**	Akuta, N, 2013 [55]	Ultra-deep sequencing	T54S; V36A; V170A	0.20%	Japan	24	Lower SVR rate
**9**	Beloukas, A, 2015 [56]	Sanger and Deep sequencing	Q80K; V36L; V55A; V36M	1%	England	20	NS
**10**	Murakami, E, 2014 [57]	Ultra-deep sequencing	Y93H	0.1–0.5%	Japan	18	Treatment failure

Keyword: NS: Not specified.

## Data Availability

The original contributions presented in this study are included in the article/Supplementary Material. Further inquiries can be directed to the corresponding author.

## Supplementary Materials

The following supporting information can be downloaded at: https://www.mdpi.com/article/10.3390/ijerph22111670/s1, File S1: Raw data used to generate figures and tables reported within the article.
